# Who Are Dentists?

**Published:** 1862-01

**Authors:** J. Allen

**Affiliations:** New York


### WHO ARE DENTISTS ?

BY J. ALLEN, D. D. S.

I DID not expect to offer any further remarks, through the medium of your journal, upon the subject of Who are Dentists; but an article in the December number of the Register, by Dr. Pease, seems to call for a passing notice, for I perceive that he still errs in his statements, notwithstanding the efforts that have been made to set him right.

This may arise, perhaps, from mere force of habit, rather than design on his part; but in the article above referred to, he is in error on several points, a few of which only will here be noticed, leaving his personal reflections upon me to pass without comment, except to correct misstatements.

First, he is mistaken as to my motive in reviewing his articles upon Who are Dentists; for he says my "object was purely selfish." Upon this point I can assure the Doctor that I have no more interest in the matter than any other

*See Koelliker's Mic. Anatomy II, 54—123 for what is new upon the subject of the teeth their literature, chemistry, caries &c. Ficinus upon the loss and caries of the teeth by Walther and Ammons Journal of Surgery &c., &c.

member of the profession. My review simply referred to statements made by him, which were at variance with the facts, some of which are as follows: He stated that "dentistry as a profession is of American origin. It had its rise in a great public want, which nowhere but in the United States existed, and there is little likelihood that it ever will exist in any other country." These declarations do not tally with the facts, to which I simply referred. Again, he says "dentistry can not be said to date back much earlier than 1840." Nor is this statement sustained by the history of our profession.

He also made other assertions, equally erroneous, which I endeavored to point out, to which he now takes exceptions, and instead of attempting to controvert my corrections, he seems to regard them as a personal affair, which I disclaim; for the fact connected with the subject concerns the whole dental profession as much as him or me.

But he says, my "object was also to discredit him as a writer." To this charge I would simply state that I have no more wish to discredit his writings, than those of any other person, provided they are truthful. Neither do I feel bound to endorse them if untrue, merely because they emanate from his pen.

Another example of his proneness to wander in his writings is apparent in the following sentence, the odium of which he now attempts to turn upon me. Dr. Pease stated: "There are two classes of dentists, or rather, there are dentists and mechanics, from whom very different treatment may be expected. The one feeling little more responsibility resting upon them than what is common to mechanics, act accordingly; they persistently seek for the sale of their wares, talk loudly and promise much; they obtrusively thrust forward their mechanical contrivances, and press pieces of artificial teeth on the attention of the public, all of which the other as studiously avoid. The one never rises above the customs of a craft, or trade; the other is governed by the rules of a profession." Surely the Doctor will recognize this as his own language; upon which I made the following comment: Although there may be some to whom the above remarks may be applied with truth, yet to speak thus disparagingly of *all* who insert artificial dentures, (and he makes no exceptions), evinces one of two things: either that the position he occupies in the profession is unfavorable to command a full view of it; or his zeal to eulogize one branch of dentistry at the expense of another has warped his better judgment.

But Dr. Pease now writes as follows: "I called the manufacturers of artificial dentures *mechanics*. Dr. Allen has seized upon that term, and exhausted his ingenuity in trying to convert it into a disparagement of that kind of labor. This is no more than I might have expected from him." The reader can readily see who is the disparager. In like manner, he also attempts to turn upon me his contradictory statements upon the subject of artificial teeth on gold plate, and charges me with design to injure him, for he now says, in reply to my previous remarks on this point: "Contra to what? *Not surely to sets of teeth on gold, for these we recommend.*" Now here the Doctor is all wrong again. He was attempting to show who were dentists, and said: "There are two classes of dentists." "The one will preserve the natural teeth; they will refuse to extract them, merely because they ache, or have a gum-boil at the roots." "The patients of the first preserve their teeth." The patrons of the other class, he said, "get a shining set of white teeth, which every one knows to be artificial, in return for which they are but imperfectly cherished, the breath becomes offensive, the mouth falls in, the nose sticks further out, the lips shorter, the lips and cheeks become wrinkled and shriveled—the cheek bones assume an unnatural prominence, and they look prematurely old." Again he says, "A set of artificial teeth is a piece of mechanism—nothing more; it is made of *light*, *frail*, *and fracturable materials;* and it is believed that the average duration of sets of artificial teeth will not exceed *six years*."

*Now* he says he did not mean teeth on gold plate; but that he meant "continuous gum, if it related to any particular kind of artificial dentures." "And yet Dr. Allen has had the effrontery and shamelessness to take this paragraph from the place where it belongs, and to which it relates, and to quote it in connection with another part of the article, describing the excellence and superiority of sets of teeth on gold, in order to disparage that kind of dentures, and nullify what had been said in favor of it."

In answer to this charge I have only to say, the disparagement was written by *himself*, not me; and that in my review I had to take his points wherever I found them. Scattered as they were, if I have succeeded in grouping them together, so that the Doctor can see the errors and inconsistencies that were apparent in his articles, I shall have accomplished at least one object I had in view, if he avoids similar errors in future. But he *now* says he did not mean teeth on gold; it was continuous gum work he referred to when speaking so disparagingly of artificial teeth, and also that class of dentists who insert them. Now let us turn to page 295 Dental Register, and see what he *did* say about continuous gum work, for his memory seems to be rather treacherous. He says: "The question will arise, what are the advantages and disadvantages of a set of teeth made with continuous gum over one made in the ordinary manner on gold plate?" "We answer the only advantage (which is of some moment) consists in melting the enamel between and around the teeth, so as to leave no space for the accumulation of food, and in some few instances, of building out beyond the plate, so as to give the mouth and cheeks a little more fullness."

The Doctor then proceeds to point out the disadvantages, and closes by saying, "Thus it will be seen that a set of teeth on a platina base is valuable for cleanliness, and occasionally for other purposes, but that it is undesirable, from its *weight*, clumsiness, greater tax on the roof of the mouth, and also from its liability to accidents, not easily repaired." Now, does this description of dentures tally with those *light, frail, and fracturable* sets of teeth described by him so graphically, where the *breath becomes offensive*, the *mouth* and *cheeks sunken*, and the looks prematurely old? No, no; the Doctor has forgotten again, as evinced by the following sentences: "Another element of value of all mechanical work is *universality* of manufacture. A piece of mechanism, however valuable, loses much of its value, if the means of repair are not always at hand, in case of an accident." "Hence it follows, that that kind of artificial teeth that can be made and easily and cheaply repaired by *all dentists*, not only of a particular locality, but by *all dentists of the country*, are the safest, and therefore the most desirable and valuable." "Such are those mounted *on gold and silver. Such are not those* mounted on other materials, as they require a peculiar apparatus for their construction and repair, not used for other materials."

Now these quotations do show clearly that the poor, miserable artificial dentures described by him were made on gold and silver plates, and by that class of dentists of which he spoke so disparagingly, and then in turn recommends this style of artificial teeth, because all this class of dentists can make and repair it.

I should not have quoted the foregoing sentences from Dr. Pease again, but from the fact of his having misconstrued my motives, denied the points here repeated, and his attempt to misrepresent me, together with a charge of unfairness in my review. If the Doctor will now carefully review his articles upon "Who are Dentists," and compare them with the facts, he will surely see his errors and inconsistencies, and if he will but profit by what was well intended, I can well afford to pass his censorious remarks by in respectful silence.

NEW YORK, Jan. 20th, 1862.

